# Three dimensional organisation of basal bodies in *Trypanosoma brucei*

**DOI:** 10.1186/2046-2530-1-S1-P54

**Published:** 2012-11-16

**Authors:** K Towers, LC Hughes, S Vaughan

**Affiliations:** 1 Oxford Brookes University, UK

## 

Cilia and flagella are assembled from a basal body and assembly and duplication is tightly regulated with the cell cycle. A new basal body (called a pro-basal body) is formed orthogonal to the old basal body during S-phase and will assemble a cilium/flagellum in the next cell cycle. The basal body and pro-basal body are connected to each other and the pro-basal body must re-orientate and disengage from the basal body before forming a cilium/flagellum. There is still a poor molecular and ultrastructural understanding of the connections between the basal bodies and how these are re-organised during basal body duplication, re-orientation and segregation. The protozoan parasite *Trypanosoma brucei* is a well established model organism in which to study cilia/flagellum structure and function. The parasite is the causative agent of a fatal disease known as African sleeping sickness and the flagellum is a key virulence factor essential for cell viability, motility and transmission. We employed cellular electron tomography and serial thin section analysis to visualise the three dimensional organisation of basal bodies in *T. brucei*. This has enabled us to determine ultrastructural detail of basal body duplication throughout the cell cycle. We have characterised the connections which exist between basal bodies at various stages of the basal body maturation cycle and we now have greater understanding of how these connections are remodelled to allow successful re-orientation followed by timely segregation. These studies provide deeper understanding of how basal body duplication is regulated.

